# Behavior-Relevant Periodized Neural Representation of Acoustic But Not Tactile Rhythm in Humans

**DOI:** 10.1523/JNEUROSCI.0664-25.2025

**Published:** 2025-10-13

**Authors:** Cédric Lenoir, Tomas Lenc, Rainer Polak, Sylvie Nozaradan

**Affiliations:** ^1^Institute of Neuroscience (IONS), UCLouvain, Brussels 1200, Belgium; ^2^Basque Center on Cognition, Brain and Language (BCBL), Donostia-San Sebastian 20009, Spain; ^3^RITMO Centre for Interdisciplinary Studies in Rhythm, Time and Motion, University of Oslo, Oslo 0318, Norway; ^4^Department of Musicology, University of Oslo, Oslo 0318, Norway; ^5^MARCS Institute for Brain, Behaviour and Development, Western Sydney University, Sydney, New South Wales 2751, Australia; ^6^International Laboratory for Brain, Music and Sound Research (BRAMS), Montreal, Quebec H3C 3J7, Canada

**Keywords:** auditory, autocorrelation, beat, EEG, rhythm, tactile

## Abstract

Music makes people move. This human propensity to coordinate movement with musical rhythm requires multiscale temporal integration, allowing fast sensory events composing rhythmic input to be mapped onto slower, behavior-relevant, internal templates such as periodic beats. Relatedly, beat perception has been shown to involve an enhanced representation of the beat periodicities in neural activity. However, the extent to which this ability to move to the beat and the related “periodized” neural representation are shared across the senses beyond audition remains unknown. Here, we addressed this question by recording separately the electroencephalographic (EEG) responses and finger tapping to a rhythm conveyed either through acoustic or tactile inputs in healthy volunteers of either sex. The EEG responses to the acoustic rhythm, spanning a low-frequency range (below 15 Hz), showed enhanced representation of the perceived periodic beat, compatible with behavior. In contrast, the EEG responses to the tactile rhythm, spanning a broader frequency range (up to 25 Hz), did not show significant beat-related periodization and yielded less stable tapping. Together, these findings suggest a preferential role of low-frequency neural activity in supporting neural representation of the beat. Most importantly, we show that this neural representation, as well as the ability to move to the beat, is not systematically shared across the senses. More generally, these results, highlighting multimodal differences in beat processing, reveal a process of multiscale temporal integration that allows the auditory system to go beyond mere tracking of onset timing and to support higher-level internal representation and motor entrainment to rhythm.

## Significance Statement

Integrating fast sensory events composing music into slower temporal units is a cornerstone of beat perception. This study shows that this ability relies critically on low-frequency brain activity, below the sensory event rate, in response to acoustic rhythm. Conversely, brain responses elicited by the same tactile rhythm exhibit higher-frequency activity corresponding to faithful tracking of the sensory event rate. Critically, the auditory-specific slow fluctuations feature an enhanced representation of the perceived periodic beat, compatible with behavior. This higher-level neural processing of rhythmic input could thus reflect internal representations of the beat that are not shared across senses, highlighting multimodal differences in beat processing. These results pave the way to explore high-level multimodal perception and motor entrainment in humans.

## Introduction

The propensity of humans to coordinate movement with musical rhythm is attributed to a close coupling of the auditory and motor systems (Chen et al., 2008; Patel and Iversen, 2014; Patel, 2024). This audiomotor coordination is thought to rely on the auditory system’s ability to integrate information at multiple timescales (Teng et al., 2016). Such multiscale temporal integration is arguably essential for human-specific social interactions such as music and dance (Patel et al., 2005; Zatorre et al., 2007) and speech (Arnal et al., 2015b; Norman-Haignere et al., 2022). Specifically, this multiscale temporal scaffolding is crucial for organizing fast time intervals composing rhythmic input into slower, behavior-relevant templates. Once mapped onto the rhythmic input, these internal templates can be experienced as periodic beats and can be used to guide motor coordination with others and the music (Large and Snyder, 2009; London, 2012; Bouwer and Honing, 2015).

Musical beat usually refers to an internal representation consisting of recurring periods, or periodic pulses, mapped onto the rhythmic input (Bouwer and Honing, 2015; London et al., 2017; Lenc et al., 2021). How this internal periodic template is mapped onto complex sensory inputs such as music is far from trivial. Indeed, the beat periodicities are often not prominent in the physical structure of the input (London, 2012; London et al., 2017; Bouwer et al., 2018), thus pointing toward higher-level neural processes underlying beat perception (Nozaradan et al., 2017a; Lenc et al., 2021, 2025). Therefore, uncovering these processes promises key insight into high-level perception and motor entrainment in humans.

Recent studies captured human brain activity using electroencephalography (EEG) in response to rhythmic inputs known to yield the perception of a periodic beat. These studies revealed that the neural representation of rhythmic inputs exhibit selectively emphasized beat periodicities, regardless of their prominence in the input (Nozaradan et al., 2017a; Lenc et al., 2020, 2023). Importantly, such “periodized” neural representation seems functionally relevant, as the enhanced beat-related periodicities in neural activity correspond to those preferentially expressed through body movements (Nozaradan et al., 2012, 2018; Lenc et al., 2018).

However, whether this neural representation of beat and the ability to move to it are auditory-specific or generalize beyond audition remains unclear. Synchronization to rhythm is generally recognized to be facilitated with audition, as compared with vision or touch (Repp and Penel, 2004; Hove et al., 2013; Gilmore and Russo, 2021). Yet, recent studies have challenged this view by showing synchronization performance close to that typically found with audition when the rhythmic input was tuned to match the sensitivity of the sensory modality being compared with audition (e.g., with visual rhythms conveyed through moving rather than static stimuli; Hove et al., 2010).

Here, we addressed the question of cross-sensory commonalities and specificities in beat processing by separately recording behavioral and EEG responses to an acoustic and tactile version of the same rhythm. Since sounds and vibrations share similar physical attributes (i.e., time–amplitude varying signals characterized by their magnitude, frequency, period, and wavelength; Dobie and Van Hemel, 2005) and often concomitantly occur in musical contexts (Merchel and Altinsoy, 2018; Reybrouck et al., 2019), it could be hypothesized to find enhanced neural representation of the beat in response to both types of sensory input (Schurmann et al., 2006; Rahman et al., 2020). Yet, recent studies on sensorimotor synchronization with tactile versus acoustic rhythms and corresponding EEG activity (Brochard et al., 2008; Tranchant et al., 2017; Gilmore and Russo, 2021) showed differences between these modalities. These differences could have been driven by the use of stimuli not specifically adjusted for the somatosensory system (e.g., body part and carrier frequency used for stimulation suboptimal to the mechanoreceptors' sensitivity). Therefore, the current study aimed to move a critical step forward by investigating beat processing using stimuli fine-tuned to match the sensitivity of each sense while controlling for lower-level confounds using rhythms whose physical structure does not feature prominent beat periodicities.

## Materials and Methods

### Participants

Forty-five healthy volunteers (32 women; mean age, 23.5 years; SD, 3.4) took part in the study. All participants reported normal hearing, no alteration of cutaneous sensitivity at the level of the fingers, and no history or presence of neurological or psychiatric disorders. Most of the participants reported having grown up in countries from a Western culture (42 out of 45). They reported a range of musical training (mean, 1.6 years of formal music training, e.g., music lessons; SD, 3.0; range, 0–14 years), with 8 of them self-identifying as “amateur musicians” while the other 37 participants defined themselves as “nonmusicians.”

Participants were randomly assigned to one of the three groups corresponding to three different block orders, i.e., Group 1, first tactile–second acoustic–third tactile (*n* = 15; mean age, 25.9 years; SD, 4.3; 12 women); Group 2, first tactile–second tactile–third acoustic (*n* = 15; mean age, 25.4 years; SD, 2.3; 12 women); and Group 3, first acoustic–second tactile–third tactile (*n* = 15; mean age, 24.7 years; SD, 3.4; 8 women). Participants provided written consent after being informed about the experimental procedures. All procedures were approved by the local ethical committee “Comité Hospitalo-facultaire de l’UCLouvain” (protocol number B403201938913).

### Rhythmic sequences

**Figure 1.**
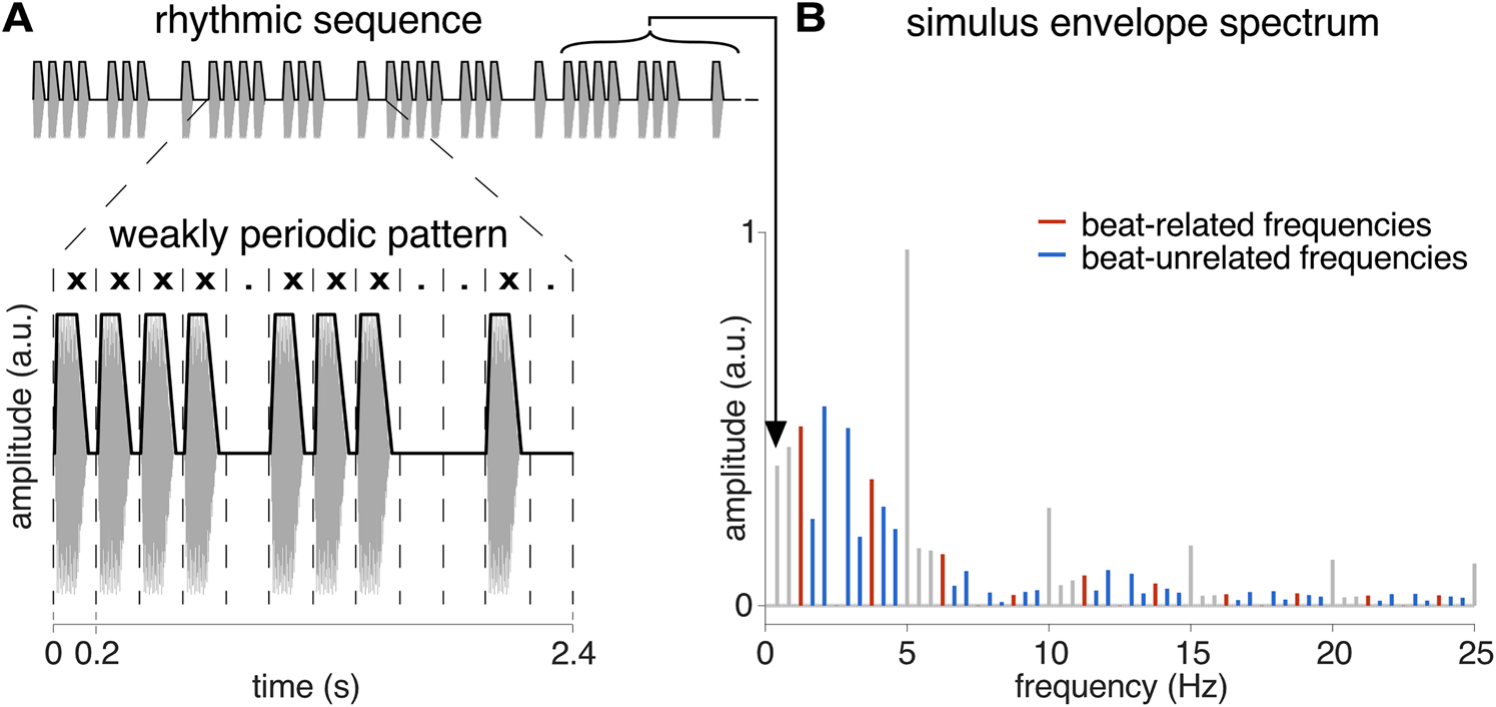
Stimuli consist of a repeated rhythmic pattern where the beat periodicity is not prominent. ***A***, Time domain representation of the stimuli (full signal in gray, sound envelope in black). The 2.4 s rhythmic pattern was looped 25 times, yielding 60 s sequences for both sensory modalities (carrier frequency of 300 Hz and 86 Hz for acoustic and tactile sequences, respectively). ***B***, Magnitude spectrum of stimulus envelope. Frequencies of interest were defined among the frequency of pattern repetition (1/2.4 s = 0.417 Hz) and harmonics. Harmonic frequencies corresponding to the most consistently tapped periods were defined as beat-related frequencies in red (4 × 200 ms = 800 ms, i.e., 1.25 Hz and harmonics) and the remaining frequencies as beat-unrelated frequencies in blue. Note that beat-related frequencies (defined as the most consistently tapped period of 800 ms, i.e., 1.25 Hz and harmonics) were overall of lower magnitude than beat-unrelated frequencies in this rhythm.

Participants were presented with 60 s rhythmic sequences in both sensory modalities. The sequences were created in MATLAB 2020b (MathWorks) by seamlessly looping 25 times a specific 2.4 s rhythmic pattern. The pattern was built by dividing 2.4 s into 12 equal intervals of 200 ms, with 8 of these intervals being allocated a sensory event kept identical across the eight occurrences. The location of these sensory events on this regular interval grid yielded a specific rhythmic pattern corresponding to [xxxx.xxx..x.] (where “x” is a sensory event and dot represents an empty grid interval, each spanning 200 ms). The sensory events corresponded to 150-ms-long sounds or vibrations (10 ms linear ramp-up, 90 ms plateau, and 50 ms linear ramp-down), followed by a 50 ms gap (see Fig. 1*A* for a visualization of the sequential arrangement of sensory events composing this specific pattern).

This rhythmic pattern, used in a number of previous studies (Nozaradan et al., 2016b, 2017a,b; Lenc et al., 2018; Sauvé et al., 2022; Sifuentes-Ortega et al., 2022), has been repeatedly shown to induce perception of a periodic pulse (beat) at a rate generally converging across Western participants toward a grouping of four underlying grid intervals (4 × 200 ms). Moreover, significant relative enhancement of neural activity at frequencies corresponding to the rate of this beat periodicity and harmonics has been observed in the EEG in responses to this repeated pattern (Nozaradan et al., 2012, 2018; Lenc et al., 2023). Importantly, this specific pattern can be considered weakly periodic at the rate of this beat, since the groups of sensory events making up the rhythm are arranged in a way that does not prominently cue the beat periodicity (Povel and Essens, 1985; Patel et al., 2005; Grahn and Brett, 2007). Using a weakly periodic rhythm is critical here, as it allows to control for low-level sensory confounds, whereby the observed neural emphasis on the beat periodicity could otherwise be trivially explained by neural responses elicited by prominent physical features of the stimulus (see below and Fig. 1*B*, for a quantification of the prominence of the beat periodicity in the stimulus modulation signal).

#### Acoustic inputs

Sound events consisted of pure sine waves at a frequency of 300 Hz. This carrier frequency was used here based on previous work having shown that stimuli within this frequency range elicit robust EEG responses (Wunderlich and Cone-Wesson, 2001; Ross et al., 2003; Nozaradan et al., 2015, 2018; Lenc et al., 2018; Rahman et al., 2020). Acoustic sequences were delivered binaurally, to ensure comparability with effects obtained in previous studies investigating beat processing using similar acoustic rhythm (Nozaradan et al., 2016b, 2017a,b; Lenc et al., 2018; Sauvé et al., 2022; Sifuentes-Ortega et al., 2022). Sounds were presented at an intensity of 70 dB SPL using flat frequency response insert earphones (ER-2, Etymotic Research).

#### Tactile inputs

Vibration events consisted of 86 Hz sine waves at an intensity corresponding to a peak-to-peak displacement magnitude of 230 µm. Previous works have shown that such tactile stimuli effectively recruit the different types of mechanosensitive receptors and elicit robust EEG responses (Muniak et al., 2007; Bensmaia, 2008; Rahman et al., 2020). Tactile sequences were generated by an electromagnetically shielded piezo-electric vibrotactile stimulator (VTS, Arsalis, UCLouvain) connected to a 20-mm-diameter round–tipped probe. Tactile sequences were delivered unilaterally to all fingertips in contact with the probe, which allowed participants to perform the tapping task with the other hand similarly to the acoustic condition while keeping tactile stimulus presentation identical across EEG and tapping sessions. The specific cutaneous region of the fingertips was chosen given its highest density of mechanoreceptors (Johansson and Vallbo, 1979; Vallbo and Johansson, 1984; Corniani and Saal, 2020) and for its distance to the ear canal which prevented any auditory response through bone or soft tissue conduction (Geal-Dor and Sohmer, 2021). Moreover, during tactile stimulation, the likelihood of eliciting auditory response to sounds produced by the piezo-electric stimulator was reduced by playing a uniformly distributed white noise through insert earphones at individually adjusted maximal tolerable intensity (up to 80 dB SPL). The masking white noise started 2 s before the onset of each tactile trial and ended 0.5 s after its end.

### Experimental design

The main experiment consisted of three blocks, one acoustic and two tactile. The tactile condition was repeated because perceiving the beat in this setting is unusual for normal-hearing individuals who are mostly exposed to and rely on auditory input in rhythmic musical contexts in everyday life. To mitigate the novelty of the situation and allow participants to familiarize themselves with the tactile condition, we repeated the tactile block and counterbalanced the order of the blocks across participants. Each block was composed of an EEG session (12 trials), followed by a tapping session (5 trials). A short break of a few seconds was included between each trial and block to prevent sensory habituation and fatigue.

During the EEG session, participants were asked to focus their attention on the tactile or acoustic rhythmic sequences and refrain from any movement. During tactile stimulation of the fingertips, the position of the forearm and wrist was comfortably stabilized by means of a cushion. To further encourage participants to focus on the temporal properties of the stimuli, participants were also asked to detect transient changes of the tempo that could possibly occur in the stimulus sequence and to report the presence and number of such changes at the end of each sequence. Tempo changes occurred in two nonconsecutive trials pseudorandomly placed within the 12 EEG trials composing each block (with the exclusion of the first trial, which never contained a tempo change). Tempo changes consisted of one pattern in which the underlying grid intervals were progressively lengthened (from 200 to 230 ms for the acoustic sequences and from 200 to 250 ms for the tactile sequences) and then shortened back to the initial 200 ms grid intervals following a cosine function across the 12 grid intervals spanning one repetition of the pattern. Within the 60 s sequences containing the tempo change, up to three nonconsecutive altered patterns were pseudorandomly positioned among the 25 repetitions of the pattern, excluding the first repetition. The altered EEG trials were removed from further analyses.

During the tapping session, participants were asked to tap the perceived beat along with the rhythmic sequences over five successive trials, using the index finger of their preferred hand. Finger tapping was recorded using a custom-build response box (hereafter “tapping box”; Institute of Neurosciences, UCLouvain) containing a high-resistance switch able to generate a trigger signal every time the fingertip contacts the response box and a force sensor continuously monitoring the normal force applied to the box (with a constant response delay of 62.5 ms). The surface of the tapping box contacted by the finger was rigid, providing somatosensory and possibly auditory feedback. This feedback was reduced by using insert earphones, which partially blocked the sound of each tap during the presentation of the acoustic stimulus and completely masked it with white noise during tactile stimulation. Participants were asked to start tapping as soon as possible after the rhythmic sequence started and continuously tap the perceived beat along with the entire sequence as regularly as possible and as synchronized as possible with the sequence. Participants were advised not to restrict spontaneous movements of other body parts if doing so would help them perform the tapping task.

### Familiarization phase

The main experiment was preceded by a familiarization phase in which the participants were briefed on the concept of (1) beat, required for the tapping task, and (2) tempo change, required for the detection task during the EEG recording sessions.

During the first part of the familiarization task, participants were asked to press the space bar of a keyboard with the index finger of their choice while listening successively to three tracks of electronic music in which the beat was acoustically either very explicit or more ambiguous. The instruction was to tap continuously along the track and as regularly as possible in synchrony with the beat they perceived, as they would do if they were nodding the head or stepping along with the music tracks. A plausible beat was indicated at the start of each music track by overlaid periodic hand-claps sounds that gradually faded out as the track progressed. Participants were asked to initially synchronize their taps to the clap sounds and keep tapping despite the cue was fading out (i.e., in a synchronization–continuation mode). Then, participants were presented with rhythmic sequences whose stimulus parameters were all identical to those used during the main experiment, except for the specific sequential arrangement of sensory events forming the repeated rhythmic pattern. Namely, we used a weakly periodic rhythmic pattern different from the one used in the main experiment, corresponding here to [xxx.xx..x.x.] (where x is a sensory event and dot an empty grid interval). These either acoustic or tactile 60 s familiarization-specific rhythmic sequences were presented, while participants were encouraged to tap along with the perceived beat as regularly as possible on the tapping box, following the exact same instructions as in the main experiment. Tactile sequences were delivered to the hand contralateral to the hand chosen by the participant to perform the tapping task. Participants were explicitly asked to relax and keep their fingers still on the stimulator while the sequences were played.

In the second part of the familiarization phase, participants were accustomed with the detection of tempo changes as occurring in a few trials of each block in the main experiment. To this aim, participants were asked to detect tempo changes pseudorandomly inserted in the familiarization-specific rhythmic sequences in the same fashion as in the main experiment.

### EEG recording and processing

The EEG was recorded using 64 sintered Ag–AgCl electrodes placed on the scalp according to the international 10/20 system (ActiveTwo, Biosemi). Two additional electrodes were placed on the left and right mastoids. The signal was referenced to the CMS (common mode sense) electrode and digitized at a 1024 Hz sampling rate (with default hardware low-pass filtering at one-fifth of the sampling rate). Electrode offsets were kept below 50 mV for all leads. The continuous EEG recordings were processed offline with Letswave6 (https://www.letswave.org/) and custom scripts in MATLAB 2020b (MathWorks). The continuous EEG signal was filtered using a 0.1 Hz Butterworth zero-phase high–pass filter (second order) to remove irrelevant slow fluctuations and then segmented into 60 s epochs relative to trial onset, thus encompassing the total duration of stimulation in each trial. Channels containing artifacts exceeding ± 200 mV or excessive noise were linearly interpolated using the three closest channels (a maximum of three channels were interpolated per participant in <7% of the total sample). After rereferencing to the common average, artifacts due to eyeblinks, eye movements, muscular activity, or heartbeat were removed using independent component analysis (FastICA algorithm; Hyvarinen and Oja, 2000). A maximum of three independent components were removed by participant. EEG responses recorded during acoustic stimulation were analyzed at a frontocentral pool of electrodes (F1, FC1, C1, F2, FC2, C2, Fz, FCz, Cz) rereferenced to the averaged mastoids which is a standard reference to estimate cortical auditory responses (Skoe and Kraus, 2010; Nozaradan et al., 2016a, 2018; Mahajan et al., 2017). EEG trials recorded during tactile stimuli were analyzed on a sensorimotor pool of electrodes contralateral to the stimulated hand (C2, C4, C6, T8, TP8, CP6, CP4, CP2, P2, P4, P6, P8) rereferenced to Fz electrode which is a standard reference to estimate cortical somatosensory responses (Tobimatsu et al., 1999; Cruccu et al., 2008; Moungou et al., 2016; Meinhold et al., 2022). EEG signals recorded during right-hand stimulation were spatially flipped over midline as if all participants were stimulated on the left fingertips. For each participant and condition, time domain EEG signals were then averaged across trials to enhance the signal-to-noise ratio of the neural response by attenuating the contribution of activities that were not time-locked to the stimulus (Mouraux et al., 2011; Nozaradan et al., 2011, 2012).

### Estimation of beat prominence in the brain responses: magnitude spectrum-based analysis

For each participant and condition, the averaged time domain EEG epochs were transformed in the frequency domain using a fast Fourier transform (FFT), yielding frequency spectra ranging from 0 to 512 Hz with a frequency resolution of 0.0167 Hz (1/60 s). A local baseline was subtracted from each frequency bin in the resulting spectrum to minimize the contribution of local variations of noise inherent to EEG recordings. The baseline was defined as the average magnitude measured at −2 to −5 and +2 to +5 frequency bins relative to each frequency bin (Mouraux et al., 2011; Retter and Rossion, 2016). Spectra were then averaged across all channels (average pool) and across modality-specific selections of channels (frontocentral pool for auditory and contralateral parietal pool for somatosensory).

### Identification of the frequencies of interest

The frequencies at which the cortical responses were expected to be elicited were determined based on the repetition rate of the rhythmic pattern. Specifically, the frequencies of interest constituted the pattern repetition rate (0.417 Hz = 1/2.4 s) and harmonics, since this set of frequencies captures any response that is reliably and consistently elicited by the repeating rhythmic pattern (Lenc et al., 2025). This set of frequencies was further validated using the temporal envelope of the 60 s sequence stimuli as computed with Hilbert transform (function “hilbert” as implemented in MATLAB 2020b). The resulting modulation signal was then transformed in the frequency domain using an FFT, yielding a frequency spectrum of the input envelope with a spectral resolution of 0.0167 Hz (Fig. 1*B*; Nozaradan et al., 2016b, 2017a). The range of included frequencies was adjusted for each modality, i.e., between 0 and 15 Hz for auditory responses and 0 and 25 Hz for somatosensory responses (see below for the identification of the response bandwidth for each modality). From the resulting set of harmonic frequencies, the first two frequencies were discarded from further analyses as they were located within a frequency range (<1 Hz) typically featuring prominent background noise in EEG spectra (due to the 1/*f*-like distribution of noise over the spectrum), thus prone to unreliable measures (Cirelli et al., 2016; Lenc et al., 2023). Moreover, the 12th frequency (i.e., 5 Hz) and its harmonics were also dismissed, as their magnitude is expected to be driven in major part by the shape of the individual 200 ms events composing the rhythmic pattern (i.e., 1/0.2 s = 5 Hz and harmonics).

### Estimation of the response frequency bandwidth for each modality

**Figure 2.**
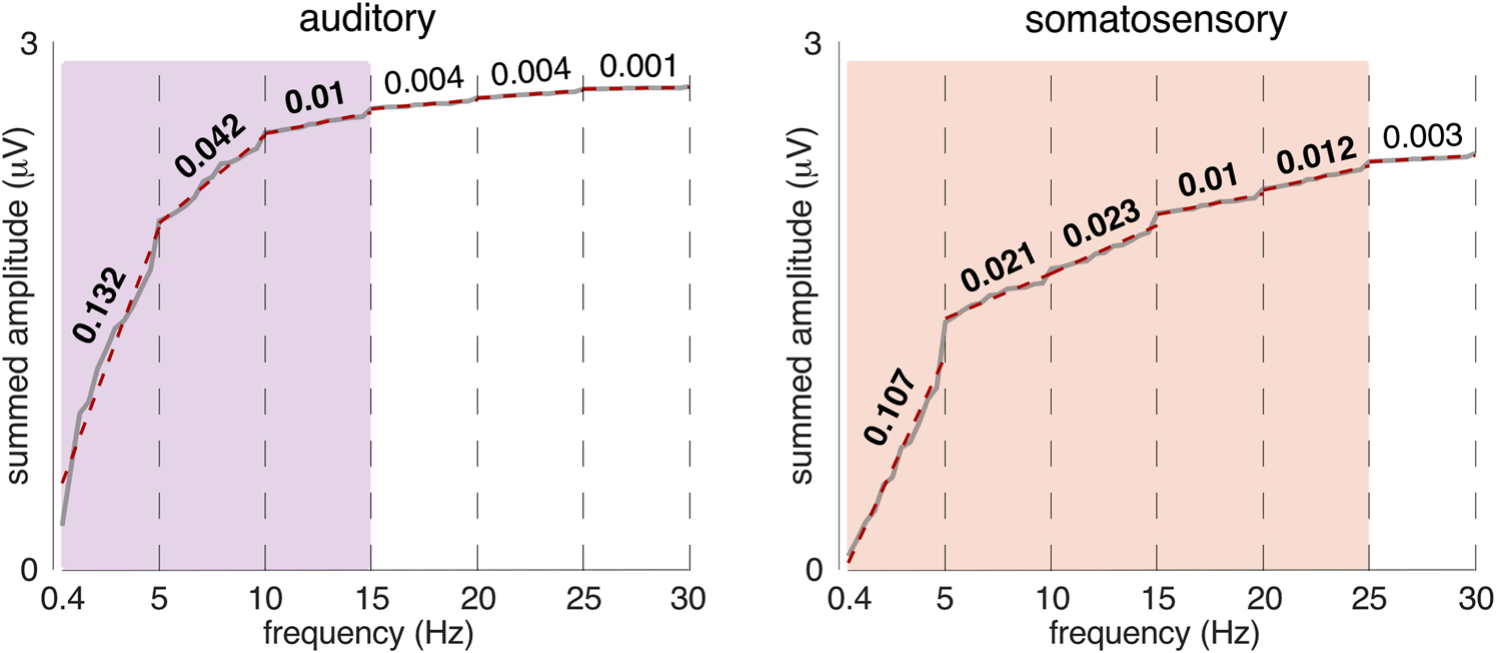
Modality-specific EEG frequency bandwidths. EEG responses show frequency bandwidths with lower cutoff for acoustic (0–15 Hz, in purple) than tactile (0–25 Hz, in green) input. The modality-specific bandwidths were determined based on the summed magnitudes over harmonic frequencies of the pattern repetition rate, measured from the group-level averaged EEG magnitude spectrum, separately for each modality. Obtained summed magnitudes (gray curve) are superimposed with red dashed lines corresponding to linear functions fitted to 5-Hz-long segments across the spectra. The slopes of the fitted lines show faster convergence toward zero for the auditory EEG responses, revealing the lower cutoff of their bandwidth. Note that most of the auditory response is concentrated below 5 Hz.

To determine the frequency range where the EEG responses for each modality were distributed, we summed the magnitudes obtained from the group-level averaged, baseline-corrected EEG spectra successively over all harmonic frequencies from 0.417 Hz (i.e., 1/2.4 s) up to 30 Hz (72 frequencies), separately for each modality. All harmonic frequencies were taken into consideration, as the aim of this analysis was to quantify the overall response to the rhythmic input irrespective of any higher-level transformation. As shown in Figure 2, the curves of the summed magnitude as a function of frequency demonstrate a substantial gain in magnitude every 5 Hz. This prominent periodicity in the EEG signals reflects the responses to the recurring shortest interonset intervals making up the rhythmic pattern (200 ms interonset or grid intervals, i.e., 5 Hz rate). We then estimated the slope of each 5 Hz segment of the obtained curves by fitting a linear regression model. Finally, the response frequency bandwidth was determined for each modality by identifying the harmonic frequency at which the slope fell below an arbitrary threshold of 0.01 (i.e., a gain in magnitude <0.05 mV over a range of 5 Hz). The estimation of the response frequency bandwidth for the auditory modality was 0–15 Hz, with most of the response concentrated below 5 Hz, while the bandwidth of the somatosensory response was 0–25 Hz. This estimation was also confirmed by computing the derivative of the curve and selecting the frequency at which the derivative was minimal and tented toward zero. Only the frequencies of interest located within the obtained response frequency bandwidths were further used to compute the beat-related *z*-scores of the stimuli and the EEG responses for each modality, respectively.

### *Z*-scored signal-to-noise ratio (zSNR) measurements

To ensure that cross-modal differences in magnitude at the frequencies of interest were not driven by different quality of the signal, zSNR was computed from the raw spectra (i.e., without baseline correction) to statistically test whether the response significantly stands out from background noise in the recorded signal (Liu-Shuang et al., 2014; Jonas et al., 2016; Lochy et al., 2018; Volfart et al., 2020; Hagen et al., 2021). First, the magnitudes at all frequency bins of interest and the respective local noise at the surrounding bins ranging from −12 to −2 and +2 to +12 were extracted. The zSNR value was then obtained as [(average magnitude across frequencies of interest) − (average baseline magnitude)] / (standard deviation of the baseline magnitude).

### Measurement of relative prominence of the beat-related frequencies

The main goal of the current study was to assess, from the whole set of frequencies of interest, the relative prominence of the frequencies considered as specifically related to the beat versus the remaining frequencies which are included in the stimulus envelope magnitude spectrum but are unrelated to the beat periodicity. To this aim, the magnitude at each of these frequencies of interest was first standardized into *z*-scores (Lenc et al., 2020). The obtained *z*-scores were then averaged separately across beat-related frequencies (4 × 200 ms = 800 ms, i.e., 1.25 Hz and harmonics) and beat-unrelated frequencies (i.e., the remaining frequencies of interest). An average beat-related *z*-score higher than the corresponding beat-related *z*-score calculated from the stimulus envelope spectrum (acoustic rhythm *z*-score = −0.059; tactile rhythm *z*-score = −0.051) would thus reflect selectively enhanced beat periodicity in the EEG compared with the input.

In addition, to assess whether the transformations observed in the EEG responses to the acoustic rhythm could not be explained by responses at the peripheral stages of sound processing, we also compared the recorded EEG responses to responses simulated with a biologically plausible model of peripheral auditory processing (hereafter cochlear model; Lenc et al., 2018, 2020). The cochlear model used to analyze the acoustic stimuli consisted in an equivalent rectangular bandwidth filter bank with 64 channels (Patterson and Holdsworth, 1996), followed by Meddis' inner hair-cell model (Meddis, 1986), as implemented in the Auditory Toolbox for MATLAB (Slaney, 1998). The output of the cochlear model was subsequently transformed into the frequency domain using FFT, and the obtained spectra were then averaged across cochlear channels. The beat-related *z*-score was then computed as described above (acoustic rhythm *z*-score from the model = −0.126, i.e., showing even less emphasis of the beat periodicities as compared with the envelope of the acoustic signal itself, as obtained with a Hilbert transform).

### Estimation of beat prominence in the brain responses: autocorrelation-based analysis

A complementary approach was applied to analyze the prominence of beat-related periodicities in brain responses using autocorrelation (Lenc et al., 2025). This novel autocorrelation-based analysis aimed to corroborate the results obtained with the magnitude spectrum-based analysis described above. Critical to the current study, the autocorrelation-based approach has the advantage of providing an estimate of periodicity that is invariant to the shape of the recurring signal. This is of major importance here, because differences in the brain responses elicited by the acoustic versus tactile sequences could be driven by characteristics unspecific to any actual beat-related periodization of the input but rather by cross-modal differences in the overall shape of the responses due to lower-level properties specific to each sensory modality. In other words, because the shape of the response to single acoustic versus tactile events is expected to differ, and because these differences would favor some frequencies in one modality and not the other, it is crucial to control this using an analysis that is insensitive to the response shape. As implemented here, the autocorrelation function (ACF) was estimated from the complex spectrum after subtracting an estimate of the 1/*f*-like noise and zeroing out frequency bins that did not correspond to the frequencies of interest. This later step is justified by the fact that the response was elicited by a stimulus consisting of a seamlessly looped rhythmic pattern and was therefore expected to elicit a response that only contains energy at the exact frequency bins corresponding to integer multiples of the rhythmic pattern repetition rate (Lenc et al., 2025), i.e., within the set of beat-related and beat-unrelated frequencies defined above (see above, Identification of the frequencies of interest). From this ACF, we extracted autocorrelation values at lags of interest corresponding to (1) beat periodicities (beat-related lags, 0.8 s and multiples from which 2.4 s corresponding to the pattern duration was excluded) and (2) control lags corresponding to periodicities where the beat was not perceived despite being compatible with the temporal arrangement of the sounds making up the rhythmic stimulus (beat-unrelated lags, 0.6, 1, 1.4, and 1.8 s and multiples). Overlapping beat-related and beat-unrelated lags were excluded. After normalizing the autocorrelation coefficients across the whole set of lags using *z*-scoring, the periodicity of the response at the rate of the beat was quantified by averaging the coefficients across beat-related lags (for further methodological details, see Lenc et al., 2025). An average beat-related *z*-score higher than the corresponding beat-related *z*-score calculated from the stimulus envelope spectrum (*z*-score = −0.409 for both acoustic and tactile rhythms) would thus reflect selectively enhanced beat periodicity in the EEG compared with the input.

### Tapping recording and analysis

The tap onsets generated by the tapping box were sent as analog triggers to the EEG system and recorded at the same sampling rate as the EEG signal (i.e., 1,024 Hz). The force signal continuously monitored by the tapping box was digitalized at 44,100 Hz and recorded by means of an audio interface (Fireface UC, RME). The time series of the continuous force signal were downsampled to 1,024 Hz after having been low-pass filtered at 300 Hz (i.e., below the Nyquist frequency of the target sampling rate) to avoid aliasing. Time series of tap onsets were converted into a continuous time domain signal with duration corresponding to the length of the stimulus sequence and sampled at 1,024 Hz. The value of each sample corresponding to a tap onset time was set to 1 (i.e., a unit impulse) and 0 otherwise.

### Circular analysis of tapping

The period of the beat perceived by the participants was determined based on an estimation of the periods that were most consistently tapped across participants. This was achieved by computing the median intertap interval (ITI) using the tap onset times (i.e., times at which the finger contacted the tapping box) for each trial, block, and participant separately. Because participants typically waited a few sensory events before starting to tap along with the rhythmic input, the first 2.4 s (i.e., temporal window of the first pattern presentation) was discarded.

To quantify the tapping performance of each participant, we then evaluated the stability of tapping with respect to the beat period (i.e., period locking) by calculating a circular measure, namely, the mean vector length (Berens, 2009; Nozaradan et al., 2016a). This measure was calculated by first selecting, based on the median ITI of each participant, the closest plausible beat period, i.e., the beat period most likely targeted by the participant (with plausible beat periods corresponding here to any integer multiple of the 200 ms grid interval that would fit within the 2.4 s rhythmic pattern, thus yielding six plausible beat periods in total: 200, 400, 600, 800, 1,200, or 2,400 ms). The obtained target beat period was then used to compute a time series of target beat positions, with phase zero set in accordance with the first tap of each trial.

The signed difference between each tap and the closest target beat position was then converted into an angle and mapped onto a unit circle. The resulting unit vectors were then averaged across trials, and the length of the mean vector served as an index of beat stability. This mean vector length thus reflected the “consistency” of asynchronies between taps and the corresponding beat positions, i.e., the strength of period locking between the beat and the tapping response (Rosenblum et al., 2001). Finally, to compare the tapping stability between blocks and participants, we obtained a single stability value per block and participant by first subtracting for each trial the angle of the mean vector from the unit vectors corresponding to individual taps, then by collapsing the obtained values across trials, and finally by recalculating the mean vector.

### Magnitude spectrum-based analysis of tap onsets and tap force

As a complementary estimate of stability in tapping to the beat, single trials of (1) time series of tap onsets and (2) continuous force signals as recorded by the tapping box were also transformed in the frequency domain using FFT. For each block and participant, and similarly to the analysis described above for stimulus input and EEG responses, *z*-scores of beat-related and beat-unrelated frequencies were then calculated from the magnitude spectrum obtained for each trial and subsequently averaged across trials. The frequency ranges of interest were defined as for EEG responses using the changes in slopes of the 5 Hz chunks obtained by successively summing the magnitude across harmonics of the pattern duration as computed on the group-level averaged magnitude spectrum for each type of signal (time series of tap onsets and tap force) and modality. Tapping responses were mainly distributed between 0 and 10 Hz for both types of signals and both sensory modalities.

### Autocorrelation-based analysis of tap onsets and tap force

The relative prominence of beat periodicities was also assessed in time series of tap onsets and tap force using the novel implementation of the frequency-tagging approach based on autocorrelation as described above for EEG responses (Lenc et al., 2025). Since tapping responses are not expected to contain a prominent 1/*f*-like noise component, the ACF was estimated directly from the raw complex spectrum.

### Head movements control

Unintentional periodic head movements of participants synchronized to the perceived beat during the EEG recording may potentially enhance beat-related periodicities in the obtained EEG responses. To rule out such artifacts, we monitored head movements by means of a two-axes accelerometer (*x* for left-right, *y* for back-front) strapped on the EEG cap at the vertex. Signals were acquired at a 1,024 Hz sampling rate. The relative prominence of beat-related frequencies was estimated in the accelerometer data in the same way as for tapping data.

### Statistical analyses

The statistical analyses were conducted using JASP (JASP Team 2023, Version 0.17.2). First, we verified that the data followed a normal distribution using Shapiro–Wilk tests. To assess if the order of blocks influenced the different measures obtained from EEG or tapping, we performed mixed repeated-measures analyses of variance (RM-ANOVAs) with “Group” as a between-subject factor (Group 1 acoustic–tactile–tactile vs Group 2 tactile–acoustic–tactile vs Group 3 tactile–tactile–acoustic) and “Block” as a within-subject factor (acoustic block vs first tactile block vs second tactile block). For between-subject comparisons, homogeneity of variance was verified using Levene’s test. Sphericity was tested using Mauchly's test and *F* values were Greenhouse–Geisser corrected when this assumption was violated. Post hoc comparisons were then performed using *t* tests, with Holm correction for multiple comparisons. In case of nonparametric testing, Friedman tests were performed with the factor “Block,” with Conover's tests used for post hoc comparisons. In addition to this frequentist analysis, we also calculated Bayes factors (BF_10_) to quantify the probability of the data under different models (Rouder et al., 2017; van den Bergh et al., 2020). Then, the inclusion Bayes factor (BF_incl_) was estimated for each model’s predictor to quantify the evidence in favor of including each of those predictors in the best model.

Finally, to assess the periodization of the EEG responses with respect to the input, we performed one-sample *t* tests between the beat-related *z*-scores of the EEG and the beat-related *z*-scores of the stimuli for each modality.

### Code accessibility

The code for the autocorrelation-based approach analysis is available at https://github.com/TomasLenc/acf_tools. Acoustic and tactile stimuli are available upon request.

## Results

### Tapping data

#### Convergent beat periodicities across modalities revealed by tapping

**Figure 3.**
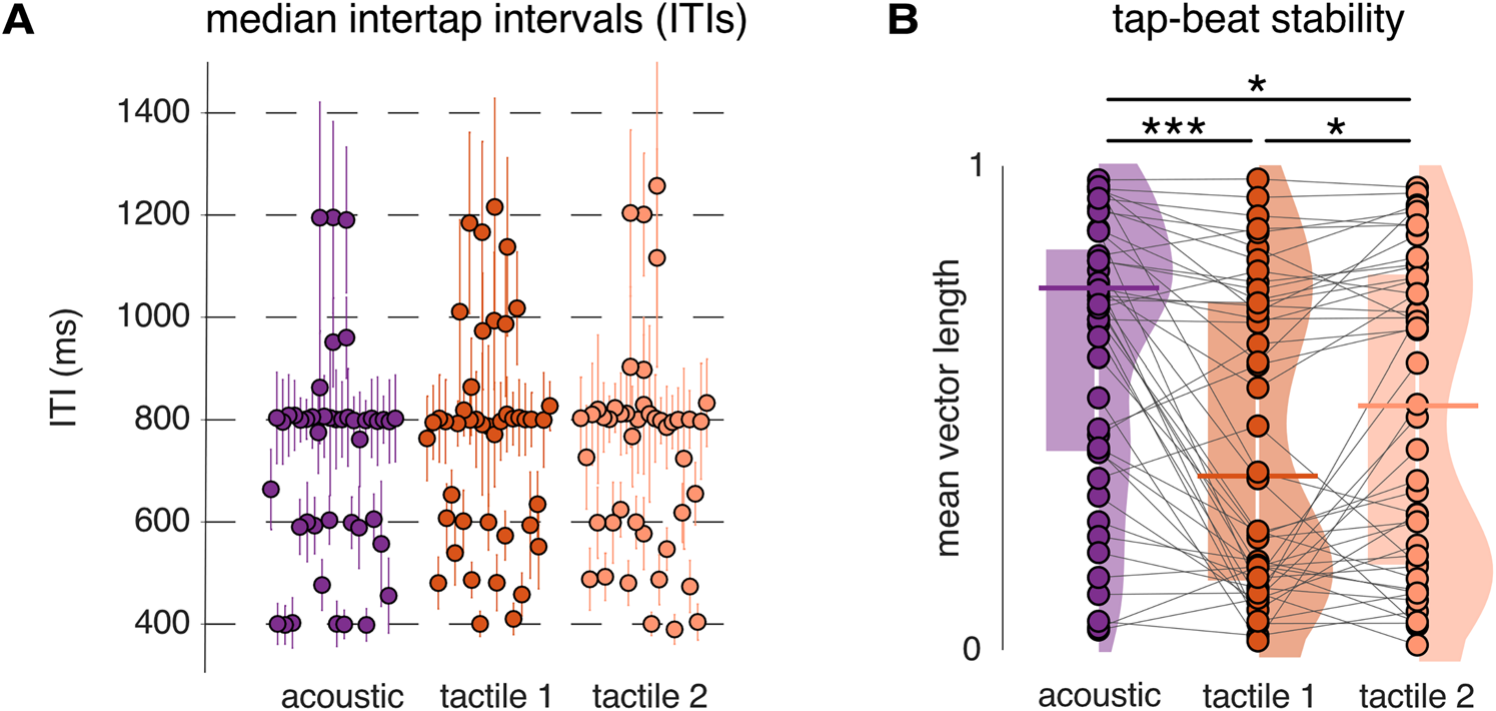
The tapping task reveals significantly lower beat tapping stability in response to the tactile versus acoustic rhythm. ***A***, Subject-level median ITIs (one dot per participant; error bars indicate interquartile ranges) for each block condition. The tapped beat periods converged toward 800 ms across all blocks (4 × 200 ms grid interval, i.e., 3 beats per pattern repetition). One data point ∼2.4 s in the three blocks is omitted from the plot for visualization purposes. ***B***, Tapping stability (one dot per participant; horizontal lines indicate the median; left-shaded boxes indicate interquartile ranges; fitted distribution densities are displayed on the right, separately for each block). Tapping stability was significantly reduced in the tactile conditions as compared with the acoustic condition (i.e., resultant vector length of the circular distribution of asynchronies between taps and beat positions closer to zero). Note that both panels ***A*** and ***B*** depict each block, irrespective of the order in which they were presented (given the absence of order effect). Asterisks indicate significant differences between blocks obtained from Conover’s post hoc comparisons (*p < 0.05; ***p < 0.001).

Median ITI indicated beat periodicities converging at 800 ms (corresponding to the grouping of four 200 ms grid intervals, i.e., three beats within each pattern repetition). Subject-level median ITIs were not significantly different between groups corresponding to different orders of block presentation (main effect of group, *F*_(2,42)_ = 0.454; *p* = 0.638; *η*_p_^2^ = 0.021; RM-ANOVA). Moreover, the median ITIs did not differ between blocks (Fig. 3*A*; main effect of block, *F*_(1.3,54.73)_ = 2.412; *p* = 0.118; *η*_p_^2^ = 0.054), and no significant Group × Block interaction was observed (*F*_(2.6,54.73)_ = 0.573; *p* = 0.611; *η*_p_^2^ = 0.027). These results were corroborated by a Bayesian RM-ANOVA which showed that the null model better explained the data than any other model including the Group and Block factors and their interaction (all BF_10_ ≤ 0.584).

#### Reduced beat periodicities in tapping to tactile versus acoustic inputs

*Beat tapping stability.* The obtained beat tapping stability as estimated with mean vector length yielded values deviating from a normal distribution (Shapiro–Wilk test, *p* < 0.002), which justified further use of Friedman tests for comparison across blocks and groups and Conover's tests for post hoc comparisons. The Friedman test revealed a significant effect of Group (corresponding to different orders of block presentation; *χ*^2^_(2)_ = 18.711; *p* = 8.648 × 10^−5^; *W* = 0.208). Conover's post hoc comparisons showed significant differences between blocks (Fig. 3*B*), namely, with larger stability for the acoustic block as compared with the first tactile (*p* = 1.218 × 10^−4^) and second tactile block (*p* = 0.048) and between the two tactile blocks (*p* = 0.045). These results were corroborated by a Bayesian RM-ANOVA which showed that the model including the Block factor (evidence for Block effect BF_incl _= 7,403.78) better explained the data than the null model or any model including the Group factor (all BF_10_ ≤ 0.366).

**Figure 4.**
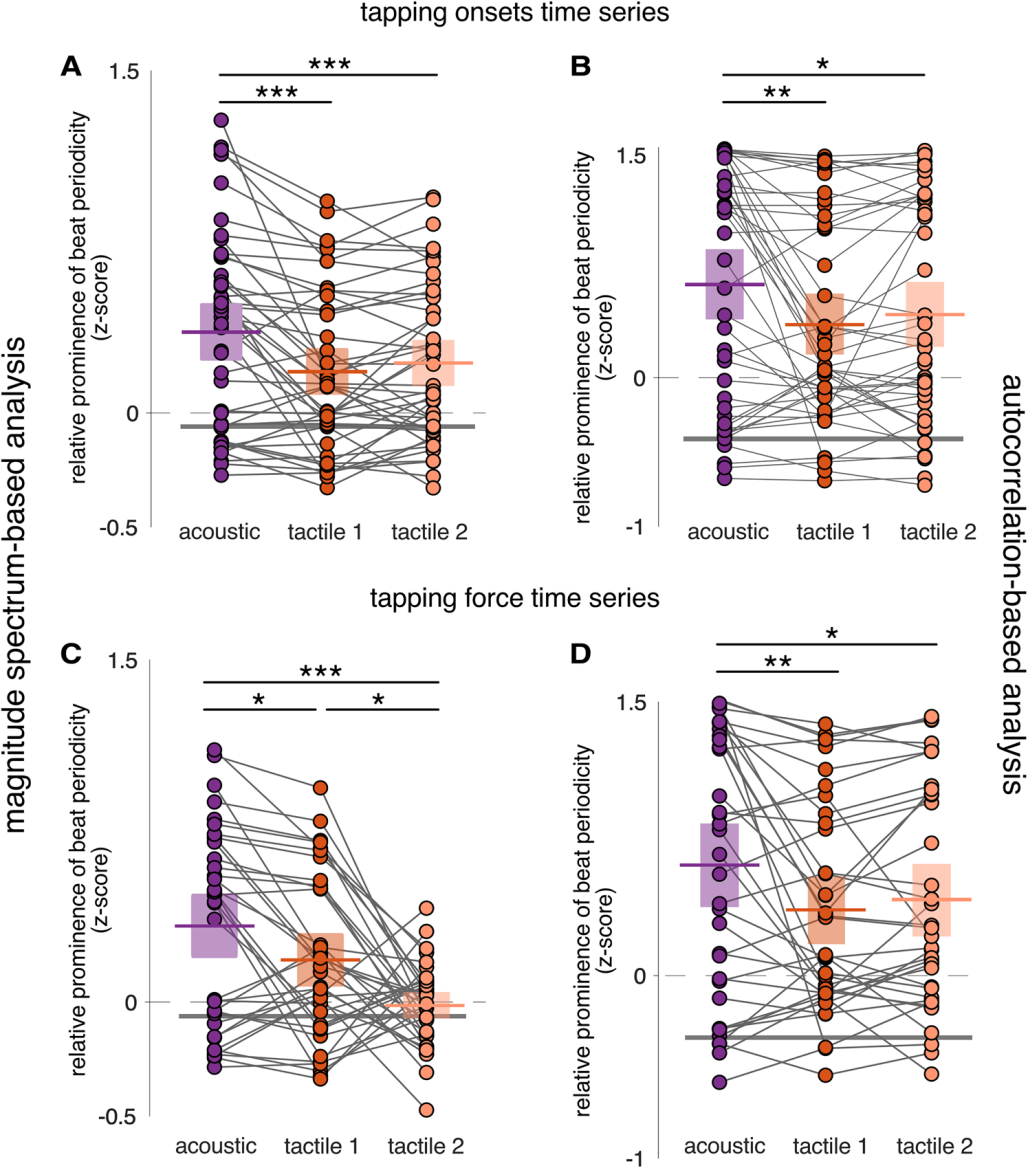
Tapping data show greater prominence of the beat periodicity in response to acoustic versus tactile rhythm. Tap onsets and tapping force are depicted in ***A*** and ***B*** and ***C*** and ***D***, respectively. Magnitude spectrum-based and autocorrelation-based analyses are depicted in ***A*** and ***C*** and ***B*** and ***D***, respectively. Note the significantly greater periodization of acoustic versus tactile input (one dot per participant; colored horizontal lines indicate means; boxes show 95% confidence intervals; gray lines for corresponding values from stimulus envelope). Asterisks indicate significant differences between blocks obtained from the pairwise post hoc comparisons (*p < 0.05; **p < 0.01; ***p < 0.001).

*Beat prominence in tap onsets time series.* The mean *z*-scored magnitudes at beat-related frequencies obtained using magnitude spectrum-based analysis corroborated the results obtained above with mean vector length. Namely, the *z*-scored values were not affected by the factor Group (*F*_(2,42)_ = 0.168; *p* = 0.846; *η*_p_^2^ = 0.008; RM-ANOVA). There was a significant effect of Block (*F*_(1.57,65.95)_ = 12.405; *p* = 1.057 × 10^−4^; *η*_p_^2^ = 0.228; RM-ANOVA), and no significant Group × Block interaction (*F*_(3.14,65.95)_ = 0.360; *p* = 0.791; *η*_p_^2^ = 0.017). Post hoc comparisons showed that beat-related frequencies were significantly more prominent in the acoustic block as compared with the first (*t*_(44)_ = 4.733; *p* = 2.663 × 10^−5^; Cohen's *d* = 0.450) and second tactile blocks (*t*_(44)_ = 3.712; *p* = 7.386 × 10^−4^; Cohen's *d* = 0.353; paired *t* test), and the two tactile blocks were not significantly different from each other (*t*_(44)_ = −1.021; *p* = 0.310; Cohen's *d* = −0.097; paired *t* test; Fig. 4*A*). These results were corroborated by a Bayesian RM-ANOVA, which showed that the best model included only the Block factor (effect of Block BF_incl_ = 1,397.7) and better explained the data than the null model or any models including the Group factor (all BF_10_ ≤ 0.379).

The results were further confirmed using beat-related *z*-scored values obtained by the autocorrelation-based analysis at beat-related lags (Fig. 4*B*). There was no significant main effect of Group (*F*_(2,42)_ = 1.0; *p* = 0.376; *η*_p_^2^ = 0.045; RM-ANOVA), a significant effect of Block (*F*_(1.49,62.43)_ = 7.487; *p* = 0.003; *η*_p_^2^ = 0.151; RM-ANOVA), and no significant Group' Block interaction (*F*_(2.97,62.43)_ = 0.572; *p* = 0.634; *η*_p_^2^ = 0.027). Post hoc comparisons showed that beat-related lags were significantly more prominent in the acoustic block as compared with the first (*t*_(44)_ = 3.721; *p* = 0.001; Cohen's *d* = 0.360) and second tactile blocks (*t*_(44)_ = 2.781; *p* = 0.013; Cohen's *d* = 0.269; paired *t* test), and the two tactile blocks were not significantly different from each other (*t*_(44)_ = −0.940; *p* = 0.350; Cohen's *d* = −0.091; paired *t* test). The frequentist statistics were corroborated by a Bayesian RM-ANOVA which showed that the best model included only the Block factor (effect of Block BF_incl _= 28.46) and better explained the data than the null model or any models including the Group factor (all BF_10_ ≤ 0.551).

*Beat prominence in force signal time series.* Due to a technical problem, the force signal was recorded in 36 participants out of 45. Nevertheless, the analyses of beat prominence using the force signal converged with those performed on tap onsets. Mean *z*-scores obtained from the magnitude spectrum-based analysis were not affected by the group order of the blocks (*F*_(2,33)_ = 0.335; *p* = 0.718; *η*_p_^2^ = 0.020; RM-ANOVA). There was a significant effect of Block (*F*_(1.68,55.43)_ = 11.768; *p* = 1.360 × 10^−4^; *η*_p_^2^ = 0.263; RM-ANOVA) and no significant Group × Block interaction (*F*_(3.36,55.43)_ = 2.106; *p* = 0.103; *η*_p_^2^ = 0.113). Post hoc comparisons showed that beat-related frequencies were significantly more prominent in the acoustic block as compared with the first tactile (*t*_(44)_ = 2.070; *p* = 0.042; Cohen's *d* = 0.399; paired *t* test) and second tactile blocks (*t*_(44)_ = 4.835; *p* = 2.495 × 10^−5^; Cohen's *d* = 0.931; paired *t* test). As compared with the analyses of onsets time series, the only noticeable difference was the significant decrease of beat-related *z*-scores in the second versus first tactile block (*t*_(44)_ = 2.765; *p* = 0.015; Cohen's *d* = 0.532; paired *t* test; Fig. 4*C*). The effect of block was supported by a Bayesian RM-ANOVA which showed that the best model included only the Block factor (effect of Block BF_incl _= 3,680.16) and better explained the data than the null model or any models including the Group factor (all BF_10_ ≤ 0.216).

The results on force time series were confirmed using the mean *z*-scored values obtained with the autocorrelation-based analysis at beat-related lags (Fig. 4*D*), also in line with the results obtained from the autocorrelation-based analysis of tap onsets (Fig. 4*B*). There was no significant main effect of Group (*F*_(2,33)_ = 1.625; *p* = 0.212; *η*_p_^2^ = 0.073; RM-ANOVA), a significant effect of Block (*F*_(1.42,46.91)_ = 6.161; *p* = 0.009; *η*_p_^2^ = 0.157; RM-ANOVA), and no significant Group' Block interaction (*F*_(2.84,46.91)_ = 0.27; *p* = 0.836; *η*_p_^2^ = 0.016). Post hoc comparisons showed that beat-related lags were significantly more prominent in the acoustic block as compared with the first (*t*_(44)_ = 3.362; *p* = 0.004; Cohen's *d* = 0.412) and second tactile blocks (*t*_(44)_ = 2.555; *p* = 0.026; Cohen's *d* = 0.313; paired *t* test), and the two tactile blocks were not significantly different from each other (*t*_(44)_ = −0.807; *p* = 0.422; Cohen's *d* = −0.099; paired *t* test). These results were corroborated by a Bayesian RM-ANOVA which showed that the best model included only the Block factor (effect of Block BF_incl_ = 7.186) and better explained the data than the null model or any models including the Group factor (all BF_10_ ≤ 0.750).

### EEG responses

#### Reduced beat periodicities in brain responses to tactile versus acoustic rhythms

**Figure 5.**
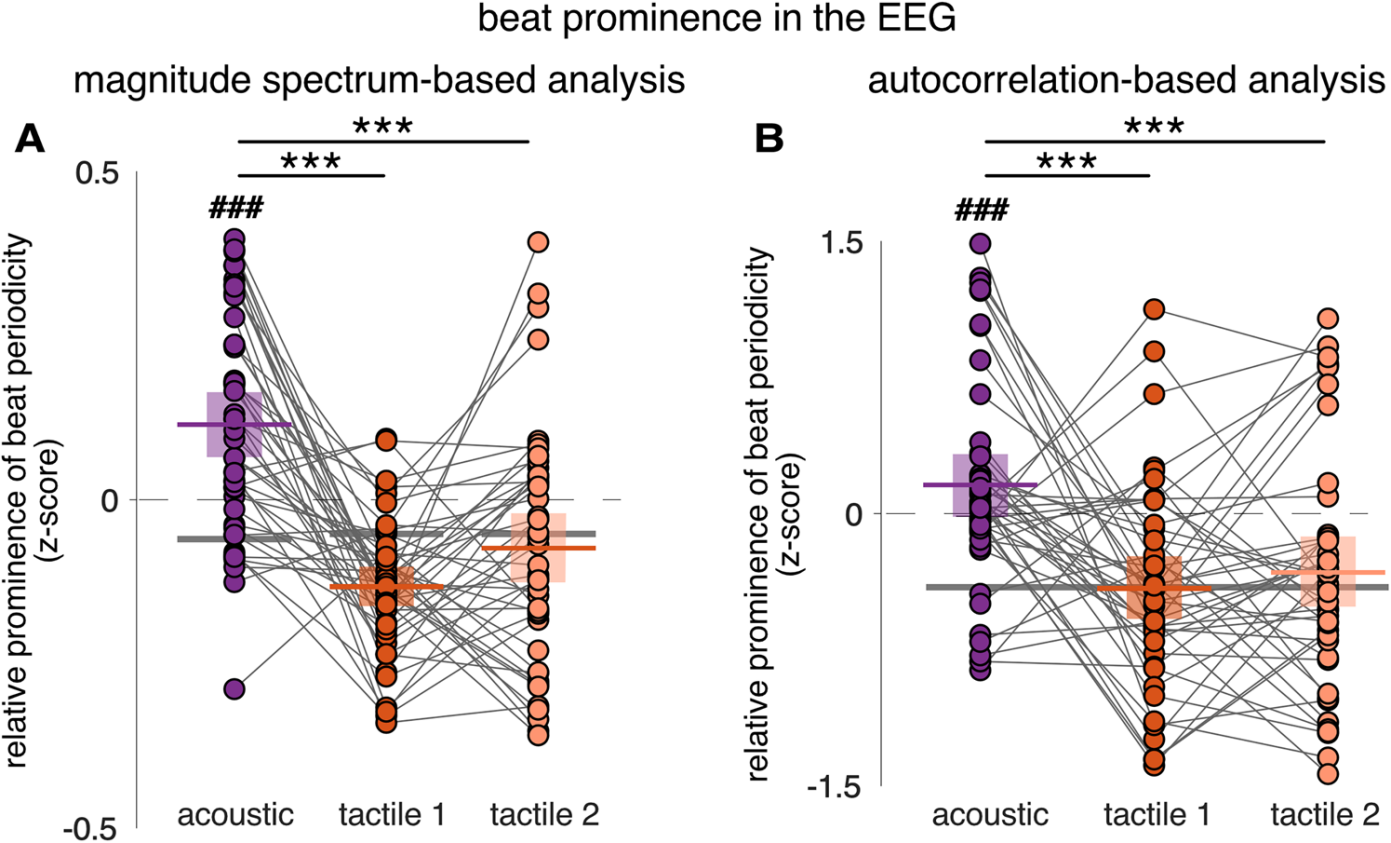
EEG responses show greater prominence of the beat periodicity for acoustic versus tactile rhythm, in line with tapping responses. ***A***, Magnitude spectrum-based analysis. ***B***, Autocorrelation-based analysis. Note the significantly reduced periodization for tactile versus acoustic inputs (cross-block and against-stimulus comparison). Horizontal lines represent means and boxes indicate 95% confidence intervals. Each dot represents a participant. The horizontal gray lines correspond to the stimuli *z*-scored magnitude at beat-related frequencies (***A***) or lags (***B***). Asterisks indicate significant differences between blocks obtained from the post hoc pairwise comparisons (***p < 0.001). The octothorpes indicate significant differences obtained from the one-sided one–sample *t* tests of the EEG *z*-scores against the stimulus *z*-scores (^###^*p* < 0.001).

The mean *z*-scored beat–related frequencies obtained using the magnitude spectrum-based approach were not affected by the group order of the blocks (*F*_(2,42)_ = 0.661; *p* = 0.522; *η*_p_^2^ = 0.031; RM-ANOVA). There was a significant effect of the Block (*F*_(1.94,81.46)_ = 37.044; *p* = 5.85 × 10^−12^; *η*_p_^2^ = 0.469; RM-ANOVA) and a significant Group × Block interaction (*F*_(3.88,81.46)_ = 3.769; *p* = 0.008; *η*_p_^2^ = 0.152). Post hoc comparisons showed that beat-related frequencies were significantly more prominent in the acoustic block as compared with the first (*t*_(44)_ = 8.240; *p* = 6.008 × 10^−12^; Cohen's *d* = 1.648; paired *t* test) and second tactile blocks (*t*_(44)_ = 6.274; *p* = 2.927 × 10^−8^; Cohen's *d* = 1.255; paired *t* test) which were not significantly different from each other (*t*_(44)_ = −1.966; *p* = 0.158; Cohen's *d* = −0.393; paired *t* test; Fig. 5*A*). These results were supported by a Bayesian RM-ANOVA which showed that the model that better explained the data included both Block and Group factors and their interaction as compared with any other models (BF_10_ ≤ 0.575). There is decisive evidence for the effect of Block (BF_incl _= 1.956 × 10^10^) followed by strong evidence for the Block × Group interaction (BF_incl_ = 10.991) and no evidence for including the Group factor (BF_incl _= 0.158).

To assess the periodization of the input in the EEG responses, one-sample *t* tests were performed between the beat-related *z*-scores of the EEG responses and the corresponding stimulus, separately for each modality. For the auditory modality, *z*-scored magnitudes of beat-related frequencies in the EEG response were significantly larger than in the stimulus beat-related *z*-score calculated from the envelope spectrum (*t*_(44)_ = 6.907; *p* = 7.818 × 10^−9^; Cohen's *d* = 1.030; one-sided one–sample *t* test against stimulus *z*-score of −0.059) and from the cochlear model (*t*_(44)_ = 9.567; *p* = 1.283 × 10^−12^; Cohen's *d* = 1.426; one-sided one–sample *t* test against stimulus *z*-score of −0.126). This was not the case in the somatosensory modality (Tactile 1 block, *t*_(44)_ = −5.258; *p* = 1.00; Cohen's *d* = −0.784; and Tactile 2 block, *t*_(44)_ = −0.815; *p* = 0.790; Cohen's *d* = −0.121; one-sided one–sample *t* tests against stimulus *z*-score of −0.051).

#### Reduced beat periodicities in brain responses to tactile versus acoustic rhythms, corroborated with autocorrelation-based analysis

Importantly, the results of beat prominence in the EEG as obtained with the magnitude spectrum-based analysis were confirmed by the autocorrelation-based analysis (Fig. 5*B*). The mean *z*-scored magnitudes at beat-related lags were not affected by the group order of the blocks (*F*_(2,42)_ = 2.263; *p* = 0.117; *η*_p_^2^ = 0.097; RM-ANOVA). There was a significant effect of Block (*F*_(1.81,76.05)_ = 12.464; *p* = 3.879 × 10^−5^; *η*_p_^2^ = 0.229; RM-ANOVA) and a significant Group × Block interaction (*F*_(3.62,76.05)_ = 2.121; *p* = 0.093; *η*_p_^2^ = 0.092). Post hoc comparisons showed that beat-related lags were significantly more prominent in the acoustic block as compared with the first tactile (*t*_(44)_ = 4.626; *p* = 4.02 × 10^−5^; Cohen's *d* = 0.960; paired *t* test) and second tactile (*t*_(44)_ = 3.940; *p* = 3.362 × 10^−4^ ; Cohen's *d* = 0.818; paired *t* test) blocks which were close to be significantly different from each other (*t*_(44)_ = −0.687; *p* = 0.494; Cohen's *d* = −0.143; paired *t* test). These results were supported by a Bayesian RM-ANOVA which showed that the model that better explained the data included the Block factor as compared with any other models (BF_10_ ≤ 0.41). Indeed, there was decisive evidence for the effect of block (BF_incl _=3,181.42), weak evidence for the Block × Group interaction (BF_incl _= 1.091), and no evidence for including the Group factor (BF_incl _= 0.376).

Similarly, as in the magnitude spectrum-based analysis, the beat-related *z*-scores obtained using the autocorrelation-based analysis showed a lack of periodization of the input in the EEG response to the tactile rhythm as opposed to the acoustic rhythm. *Z*-scored magnitudes of beat-related frequencies in the EEG response to the acoustic rhythm were significantly larger than in the stimulus *z*-scores obtained from the envelope spectrum (*t*_(44)_ = 6.495; *p* = 3.156 × 10^−8^; Cohen's *d* = 0.968; one-sided one–sample *t* test against stimulus *z*-score of −0.409) and from the cochlear model (*t*_(44)_ = 6.76; *p* = 1.288 × 10^−8^; Cohen's *d* = 1.008; one-sided one–sample *t* test against stimulus *z*-score of −0.432). This was not the case for EEG responses to the tactile rhythm (Tactile 1 block, *t*_(44)_ = −0.004; *p* = 0.502; Cohen's *d* = −6.544 × 10^−4^, and Tactile 2 block, *t*_(44)_ = 0.848; *p* = 0.201; Cohen's *d* = 0.126; one-sided one–sample *t* tests against stimulus *z*-score of −0.409).

### Additional control analyses

To control for potential biases in our analyses, we conducted several additional analyses.

#### Ruling out confounds with overall magnitude of the EEG responses

To ensure that any differences observed between conditions were not trivially explained by differences in the overall magnitude of the responses irrespective of any beat-related periodization of the input, zSNR were computed for each block by pooling over magnitudes at all frequencies of interest (i.e., including all frequencies tagged as beat-related and beat-unrelated, within the response frequency bandwidth specific to each modality). The obtained zSNR values significantly deviated from a normal distribution (Shapiro–Wilk test, *p* ≤ 0.029). The Friedman test revealed a significant effect of block (*χ*^2^_(2)_ = 10.711; *p* = 0.005; *W* = 0.119). Conover's post hoc comparisons showed significant differences between the acoustic and first tactile blocks (*p* = 0.005) and no significant difference between acoustic and second tactile blocks nor between tactile blocks (*p* = 0.153). Therefore, the differences in overall signal magnitude could not directly explain the cross-modal differences in beat-related periodization observed in the EEG data.

#### Excluding contribution of unintentional head movement artifacts in EEG responses

To rule out the possibility that beat-related periodization of the input in the EEG was driven by artifacts related to unintentional head movements of participants during the EEG recording, we estimated the prominence of beat periodicities in the accelerometer data (averaged across the two accelerometer axes) during EEG recording using the magnitude spectrum-based analysis. Due to a technical problem, accelerometer data were available for 41 participants out of 45. We compared the obtained *z*-scored magnitude of beat periodicities in the head movements to the corresponding values from the stimulus. There was a significant increase of beat periodicities in each block: acoustic (*t*_(40)_ = 2.704; *p* = 0.010; Cohen's *d* = 0.420; one-sided one–sample *t* test against stimulus *z*-score of −0.126), first tactile (*t*_(40)_ = 3.306; *p* = 0.002; Cohen's *d* = 0.516; one-sided one–sample *t* test against stimulus *z*-score of −0.051), and second tactile (*t*_(40)_ = 2.865; *p* = 0.007; Cohen's *d* = 0.447; one-sided one–sample *t* test against stimulus *z*-score of −0.051). However, there was no significant main effect of Block (*F*_(1.65,62.69)_ = 0.255; *p* = 0.733; *η*_p_^2^ = 0.007; RM-ANOVA), Group (*F*_(2,38)_ = 1.045; *p* = 0.362; *η*_p_^2^ = 0.052; RM-ANOVA), or Block × Group interaction (*F*_(3.3,62.69)_ = 1.741; *p* = 0.163; *η*_p_^2^ = 0.084; RM-ANOVA). Therefore, the unintentional head movements produced during EEG recordings were unlikely to explain the cross-modal differences in beat-related periodization observed in the EEG.

#### Modality-specific versus common average montages for EEG analyses

To ensure that the choices of the modality-specific pools of EEG channels and corresponding rereferencing did not bias the results, the same magnitude spectrum-based analysis of beat prominence in the EEG responses was performed on the signal extracted from all EEG channels rereferenced to the common average for both modalities. The results converged with the analyses performed on signals obtained from modality-specific montages. There was a significant main effect of Block (*F*_(1.79,75.29)_ = 38.586; *p* = 1.299 × 10^−12^; *η*_p_^2^ = 0.479; RM-ANOVA), no significant main effect of Group (*F*_(2,42)_ = 0.082; *p* = 0.921; *η*_p_^2^ = 0.004; RM-ANOVA), and no significant Block × Group interaction (*F*_(3.58,75.29)_ = 0.198; *p* = 0.924; *η*_p_^2^ = 0.009; RM-ANOVA).

## Discussion

The current study shows that mapping periodic beats onto an acoustic rhythm is related to enhanced beat periodicities in the neural representation of the rhythm. Moreover, this neural enhancement of the beat selectively projects onto a low-frequency range (under 15 Hz, mainly under 5 Hz). In contrast, presenting the same rhythm through the somatosensory modality does not produce such periodic neural enhancement, despite significant and comparably robust neural responses to the tactile rhythm. Importantly, this cross-sensory difference converges with differences in the ability to tap the beat along with the acoustic versus tactile rhythm.

In sum, internal representation of a rhythm that might be experienced as a periodic beat seems preferentially supported by periodized low-frequency neural activity. However, these higher-level neural representations are not necessarily shared across the senses. Such periodized low-frequency neural activity may thus reflect temporal integration across multiple timescales beyond onset timing, a distinctive specialization of the auditory system, that supports higher-level internal representation and motor coordination with rhythm.

### Periodized neural and behavioral representation of acoustic versus tactile rhythm

Most studies that investigated rhythm perception and sensorimotor synchronization across the senses have focused on instances where synchronization was meant to be performed in a one-to-one manner with the rhythmic input (Ammirante et al., 2016; Tranchant et al., 2017; Gilmore and Russo, 2021). In other words, the goal was to synchronize each movement with the onset of each sensory event. In contrast, moving to the perceived beat along with the rhythm used here, where the beat is not prominently cued, necessitates going beyond such a one-to-one mapping. Namely, it requires an internal representation showing higher degree of invariance with respect to the temporal structure of the rhythmic input. Notably, this phenomenon is not a peculiarity of experimental design constraints or of specific music genres but abounds in music worldwide (Butler, 2006; London, 2012; London et al., 2017; Witek, 2017; Câmara and Danielsen, 2018).

In the current study, the neural responses to the acoustic—but not tactile—rhythm show a specific, behaviorally relevant, enhancement of the beat period, for rhythmic inputs with a weak prominence of that beat period. This result adds to the growing evidence showing that this kind of neural enhancement could reflect internal templates of periodic beats, beyond lower-level sensory confounds (Tal et al., 2017; Nozaradan et al., 2017a; Lenc et al., 2021).

Another important observation is that a vast majority of participants spontaneously tap the beat at convergent periods, whether the rhythm is acoustic or tactile. However, while the tapping period is shared across sensory modalities, the stability in tapping the beat period is significantly lower for tactile versus acoustic rhythm. This lower tapping stability, together with the lack of emphasis of the beat period in the EEG activity, suggests a functional link between the two measures. Yet, neural and behavioral observations offer a window onto different processes recruited over very contrastive tasks—namely, experiencing a rhythm while being instructed to stay still versus actively producing synchronized movements—which might differently affect the nature of the underlying internal representations emerging during each of these tasks, respectively (Su and Pöppel, 2012; Manning and Schutz, 2013). Nonetheless, while plausibly linked, these measures do not capture the underlying processes in a strict one-to-one fashion, thus highlighting their complementarity in understanding beat perception.

### Low-frequency neural activity supports periodization of acoustic but not tactile rhythm

In the current study, neural response to the acoustic rhythm selectively projects onto the low-frequency range, mainly under 5 Hz. In the time domain, this low-frequency activity manifests as slow fluctuations punctuated by more transient responses to each sensory event onset (Fig. 5). This low-frequency activity could be a feature enabling the auditory system to integrate fast incoming events into slower, behavior-relevant, temporal units, which is critical to further coordinate body movement with rhythmic inputs. Importantly, this observation is in line with the crucial role of delta (<4 Hz) and theta (4−8 Hz) band oscillations in subserving multiscale temporal integration of temporally structured input such as music and speech in humans (Doelling et al., 2014; Arnal et al., 2015a; Teng et al., 2016, 2018) and nonhuman primates (Lakatos et al., 2005, 2016; Schroeder and Lakatos, 2009).

In contrast to the acoustic rhythm, the tactile rhythm elicits neural activity encompassing a wider frequency range, with responses mainly concentrated at 5 Hz and harmonics up to 25 Hz (i.e., harmonics of the 200 ms grid interval period). In the time domain, this higher-frequency activity takes the form of short transient responses faithfully tracking each sensory event onset and returning to the baseline before the next onset occurs. Such a faster timescale is in line with the preferential response bandwidth of 20–30 Hz previously reported for somatosensory evoked responses (Tobimatsu et al., 1999; Vlaar et al., 2015; Ahn et al., 2016). More generally, this faster response could reflect a more discrete processing of incoming tactile inputs (de Haan and Dijkerman, 2020), to the detriment of temporal integration over longer timescales.

### Capturing neural activity compatible with primary auditory and somatosensory cortices

**Figure 6.**
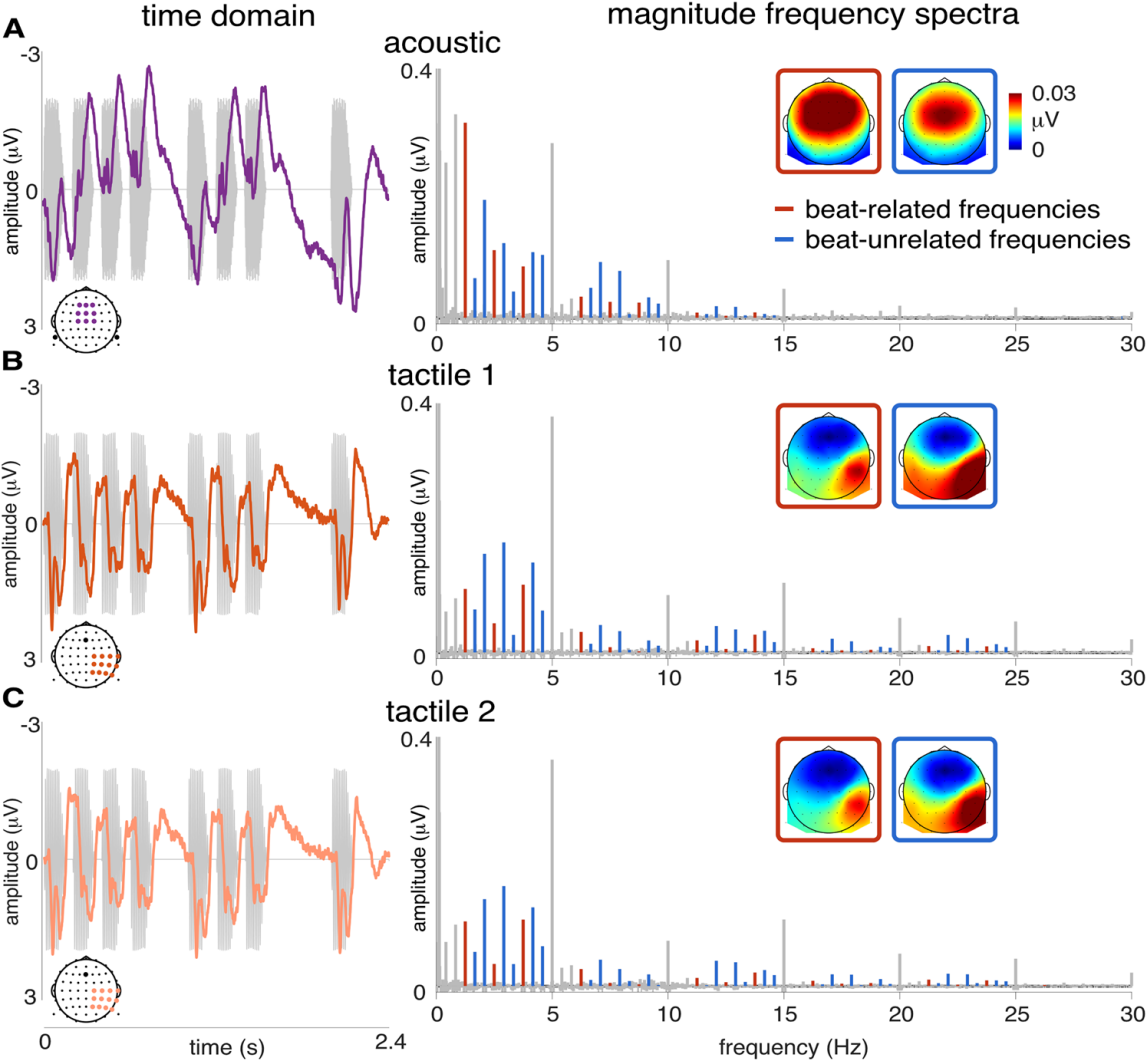
EEG responses in the time (left) and frequency domains (right) show slower fluctuations (lower-frequency) in response to the acoustic rhythm and more transient (higher-frequency) responses to tactile rhythm. Grand-average EEG responses in the acoustic (row ***A***; frontocentral pool of electrodes referenced to the averaged mastoids), and first and second tactile blocks (rows ***B*** and ***C***, respectively; sensorimotor pool of electrodes contralateral to the stimulated hand referenced to Fz). The response time course is averaged across all repetitions of the rhythmic pattern making up the stimulus sequences. Note that the auditory response time course shows transient responses to single sensory events composing the pattern (in gray), embedded into slow fluctuations of the signal. In contrast, somatosensory responses do not exhibit such slow fluctuations. On the right, the grand-average magnitude spectra show magnitude of the auditory response located within 0–15 Hz and mainly below 5 Hz, and magnitude of the somatosensory response spread over 0–25 Hz (beat-related and beat-unrelated frequencies in red and blue, respectively), in line with the estimated modality-specific EEG response frequency bandwidths displayed in Figure 2. Inserts show scalp topographies of the averaged magnitude at beat-related and beat-unrelated frequencies within each modality-specific frequency bandwidth.

The current study captured neural responses to rhythm with topographical distributions indicative of different contributions of cortical areas (Fig. 6). More specifically, the scalp topography of the somatosensory response is qualitatively compatible with activity originating from cortical generators with a dipole axis tangential to the scalp and perpendicular to the central sulcus in the hemisphere contralateral to the stimulated hand, i.e., primary somatosensory cortex (S1) generators (Allison et al., 1989; Moungou et al., 2016).

The frontocentral topographical distribution of the auditory response is known to mainly reflect activity originating from bilateral Heschl’s gyri, i.e., A1 generators (Pantev et al., 1988; Picton, 2011; Tan et al., 2016). However, such a topography does not itself rule out substantial contributions of other median brain regions (Mouraux and Iannetti, 2009; Somervail et al., 2021). Nevertheless, based on recent evidence for significant neural enhancement of the beat period observed in the human Heschl’s gyrus (Nozaradan et al., 2017a; Lenc et al., 2023), it can be reasonably assumed that A1 is embedded into a brain network enabling this higher-level temporal integration and ultimately yielding beat-related periodization of rhythmic input.

Similarly, S1 is also embedded in a functional network which comprises higher-order associative and motor areas such as the cingulate cortex and supplementary motor area, as evidenced by studies assessing temporal integration of tactile input in the context of working memory tasks (Harris et al., 2002; Numminen et al., 2004). Importantly, these studies revealed a gradient of temporal integration from S1 to these higher-order brain regions, with the latter specifically engaged into slower, suprasecond scales while the former would be crucial for retaining information in the subsecond range. In the present study, the somatosensory response to the tactile rhythm is compatible with activity predominantly originating from S1, which might thus explain the relative lack of temporal integration at longer timescales and associated reduced ability to move to the beat compared with the auditory modality.

### Multisensory redundancy in rhythm processing: somatosensation as a special case?

When parameters of rhythmic stimuli are tuned to match the sensitivity of the sensory modality, acoustic and visual rhythms have been reported to elicit comparable synchronization performances (Su and Pöppel, 2012; Gan et al., 2015). As shown here, this is not the case for tactile rhythmic stimuli. In other words, there seems to be a greater similarity in rhythm processing between the auditory and visual modalities, as compared with the somatosensory modality. This difference could be partially explained by the fact that both audition and vision share the property of being possibly stimulated by external inputs from a large distance from the body and from a remote region of the peripersonal space (Macaluso and Maravita, 2010; Canzoneri et al., 2012). The auditory and visual modalities are thus more likely stimulated concomitantly by a single external source of rhythmic inputs, particularly when positioned at distance from the body, leading to higher probability for redundancy between the auditory and visual modalities in processing rhythmic inputs.

In contrast, the somatosensory modality requires closer proximity with the stimuli for humans to perceive them. In addition, and in contrast to audition and vision, somatosensation and associated functions (such as body perception and ownership) are supported by several afferent subsystems (tactile, proprioceptive, interoceptive, vestibular; de Haan and Dijkerman, 2020) whose integration might thus be key to elicit higher-level perceptual experience such as the beat. Beat perception related to tactile input could also be influenced by the body part where the sensations are perceived. The ribcage—a location often reported as being sensitive to vibrations induced by sound (Merchel and Altinsoy, 2014)—could represent an ecologically valid alternative to the fingers stimulated here yet coming with concurrent auditory activation through bone conduction. Relatedly, it could be speculated that loud low-frequency sounds and concomitantly produced vibrations could constitute a functionally relevant input to this multientry system (Hove et al., 2020; Cameron et al., 2022). Future research is also needed to clarify how individual experience (long-term music practice or deafness) might tune the somatosensory system toward multiscale temporal integration.
